# Short-Term Outcomes following “Switching” to Monthly Ranibizumab in Neovascular Age-Related Macular Degeneration Showing Insufficient Response to Bimonthly Aflibercept

**DOI:** 10.1155/2021/5547686

**Published:** 2021-08-12

**Authors:** Jong Suk Lee, Hyun Goo Kang, Christopher Seungkyu Lee, Se Joon Woo

**Affiliations:** ^1^Department of Ophthalmology, Seoul National University College of Medicine, Seoul National University Bundang Hospital, Seongnam, Republic of Korea; ^2^Nune Eye Hospital, Seoul, Republic of Korea; ^3^Department of Ophthalmology, Institute of Vision Research, Gangnam Severance Hospital, Yonsei University College of Medicine, Seoul, Republic of Korea; ^4^Department of Ophthalmology, Institute of Vision Research, Severance Eye Hospital, Yonsei University College of Medicine, Seoul, Republic of Korea

## Abstract

**Results:**

CRT and logMAR VA were 349.62 ± 223.51 *μ*m and 0.50 ± 0.23 at the baseline and 274.69 ± 148.77 *μ*m and 0.46 ± 0.24, 311.54 ± 192.90 *μ*m and 0.45 ± 0.20 at 1 month after the first and third ranibizumab injections, respectively. The CRT decrease during three ranibizumab injections was statistically significant (38.08 ± 69.52 *μ*m, *p*=0.033). Change in VA was not statistically significant. The percentage of eyes with SRF was 100% at baseline and 53.8%, 76.9%, and 69.2% one month after each ranibizumab injections. The percentage of eyes with IRF was 38.5% at baseline and 23.1%, 23.1%, and 15.4%, respectively, after switching.

**Conclusion:**

Switching to monthly ranibizumab in nAMD showing an insufficient response to bimonthly aflibercept led to immediate anatomical improvement. It can be considered in countries where the healthcare insurance system limits the minimum injection interval of aflibercept.

## 1. Introduction

Age-related macular degeneration (AMD) is one of the significant causes of blindness worldwide. The gold standard treatment for neovascular age-related macular degeneration (nAMD) is an intravitreal antivascular endothelial growth factor (VEGF) therapy. Intravitreal anti-VEGF medications bevacizumab and ranibizumab are first introduced and recognized for improving visual and anatomical outcomes in patients with nAMD [1, 2]. Aflibercept was introduced several years later. The calculated duration of the effect of a single intravitreal injection of 2 mg aflibercept was reported to be 48–83 days due to its higher affinity for VEGF and longer half-life compared with both bevacizumab and ranibizumab [3, 4]. Monthly or bimonthly intravitreal aflibercept injections demonstrated similar therapeutic effects as monthly intravitreal ranibizumab injections [5]. Based on these reports, the health insurance system in many countries, including Korea, sets a minimum intravitreal injection interval of 2 months for aflibercept.

However, in some patients, the intravitreal injection effect of aflibercept is not maintained for 2 months. Fauser et al. [6] reported that the intraocular VEGF concentrations were suppressed below the lower limit of quantification (4 pg/mL) after intravitreal aflibercept injections for about 10 weeks on average. However, this study showed that the VEGF suppression effect by aflibercept could be shorter than two months in some patients. The real-world data of treatment and extend (T&E) treatment for nAMD using aflibercept also shows a subgroup of patients who need treatment at intervals shorter than two months [7]. As a result, more frequent anti-VEGF treatment is necessary for these patient groups. In South Korea, where the solitary national health insurance system has limited the minimum interval of aflibercept treatment to two months, switching to monthly ranibizumab treatment or additional bevacizumab treatment might be required for these patients. In most other countries, the health insurance system is not as simple as that of Korea, and the policies of several private health insurance companies are diverse and constantly changing. However, previous studies using real-world data confirmed that, in many countries, the minimum dosing interval of aflibercept is limited to 2 months. Europe, especially the United Kingdom, is a representative example (SAFARI study) [8].

Although there have been several reports about the therapeutic effect of switching from ranibizumab to aflibercept [9–14] or switch-back to ranibizumab [15, 16], data on switching from bimonthly aflibercept to monthly ranibizumab are sparse. Although most previous studies have reported favorable outcomes after switching from ranibizumab to aflibercept, it is possible that the disease's natural course and “anti-VEGF resistance” were involved in addition to the drug's effects.

This report evaluates the switching effect in terms of “anti-VEGF resistance” and the efficacy of monthly ranibizumab treatment in patients with nAMD showing an insufficient treatment response to bimonthly aflibercept; we investigated the anatomical and functional outcomes after switching from bimonthly aflibercept to monthly ranibizumab.

## 2. Materials and Methods

### 2.1. Participants

Eligible patients were ≥50 years of age with nAMD who visited Seoul National University Bundang Hospital and Severance Eye Hospital. The patients had a macula-involving active choroidal neovascularization lesion secondary to typical AMD or polypoidal choroidal vasculopathy (PCV) in the study eye. Insufficient responders to aflibercept were defined as those with subretinal fluid remaining or increasing despite more than three successive injections of aflibercept (2.0 mg/0.05 ml) at 1- or 2-month intervals, and the last injection was performed bimonthly before switching to ranibizumab. As a result, eligible patients who received three consecutive successive injections of aflibercept injections immediately before switching were enrolled, regardless of the treatment history (other anti-VEGF injections or photodynamic therapy (PDT)). In all enrolled patients, dry-up of SRF was observed at least once after initiation of anti-VEGF treatment for nAMD, and the SRF recurred while maintaining bimonthly aflibercept treatment.

### 2.2. Study Design

**Figure 1:**
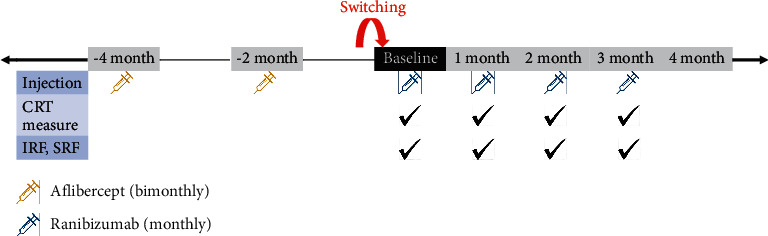
Flow diagram showing the schedule of intravitreal injections and OCT measurements of the enrolled eyes with nAMD. CRT, central retinal thickness; IRF, intraretinal fluid; SRF, subretinal fluid.

Three consecutive ranibizumab (0.5 mg/0.05 ml) doses were administered by monthly intravitreal injection at “baseline” (the day determined to “switch,” 2 months after the last aflibercept injection), “1 month” (30–37 days after first ranibizumab injection), and “2 months” (30–37 days after second ranibizumab injection). Visual acuity (VA) and optical coherence tomography (OCT) measurements were conducted at baseline, 1 month, 2 months, and 3 months (30–37 days after the third ranibizumab injection; Figure 1). From 3 months after switching (1 month after third ranibizumab injection), one of the following treatment methods was applied to each patient according to the monthly ranibizumab treatment response: maintaining monthly ranibizumab treatment, ranibizumab treatment with an extended injection interval, or reswitching to an aflibercept-based treatment (bimonthly aflibercept or monthly aflibercept or monthly cross-injection with bevacizumab).

### 2.3. Efficacy Assessments

The main outcome measurements (primary efficacy variables) included VA and central retinal thickness (CRT) using spectral-domain optical coherence tomography (SD-OCT, Spectralis OCT; Heidelberg Engineering, Heidelberg, Germany). The secondary efficacy variables included the proportion of eyes with remnant or recurrent intraretinal fluid (IRF) and subretinal fluid (SRF) to confirm the patient's retinal morphology before and after switching.

### 2.4. Statistical Analysis

To evaluate the anatomical and functional efficacy of three monthly ranibizumab treatments, CRT and VA of “1 month” and “3 months” were compared to “baseline.” Due to the small number of patients and nonnormal distribution of the observed data for the CRT and VA, the Wilcoxon signed-rank test was used. Statistical significance was defined as a *p* value < 0.05. All statistical analyses were performed using SPSS version 24.0 (SPSS Inc., Chicago, USA).

## 3. Results

We identified 13 eyes from 13 patients with neovascular AMD (including 6 eyes of PCV) showing an insufficient treatment response to aflibercept treatment. These patients showed remaining or increasing SRF during three consecutive successive aflibercept intravitreal injections before switching and received three consecutive monthly ranibizumab intravitreal injections. Of the 13 eyes, three cases were included in which the interval between the first 2 out of 3 consecutive aflibercept injections was 1 month. The demographics and treatment history of the enrolled patients are summarized in Table 1.

### 3.1. Primary Efficacy Variables

**Figure 2:**
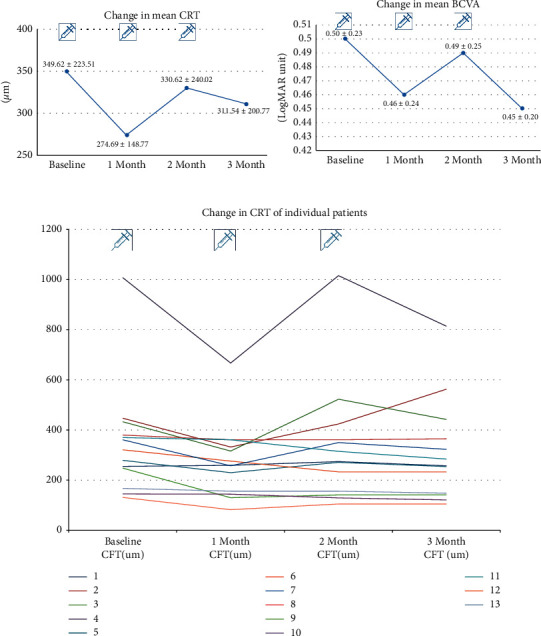
Changes in central retinal thickness (CRT) and visual acuity (VA) after switching from bimonthly aflibercept to monthly ranibizumab. The figures on the top show the change in average central retinal thickness (CRT) and best-corrected visual acuity (BCVA). The bottom figure shows the change in CRT in individual patients.

The average CRTs at baseline and 1 month after each ranibizumab injection were 349.62 ±223.51 *μ*m, 274.69 ± 148.77*μ*m, 330.62± 240.02 *μ*m, and 311.54 ± 200.77 *μ*m, respectively. The differences between the average CRT at baseline and average CRT at 1 month and 3 months (1 month after third ranibizumab injection) after the switching were statistically significant (74.92 ± 88.74 *μ*m, *p*=0.002 and 38.08 ± 69.52 *μ*m, *p*=0.033, Wilcoxon signed-rank test).  Figure 2 shows changes in individual and in average CRT and average BCVA. The average CRT most remarkably decreased 1 month after the switch and slightly increased for the second month. The differences in CRT between 1 and 2 months and 2 and 3 months were not statistically significant (*p*=0.120 and 0.074, respectively). The treatment response based on CRT worsened in two patients three months after the switching, and transient aggravation after the second ranibizumab injection was observed in eight patients.

The average LogMAR VA at baseline and 1, 2, and 3 months were 0.50 ± 0.23, 0.46 ± 0.24, 0.49 ± 0.25, and 0.45 ± 0.20, respectively. The average VA tended to improve after switching, but the average VA difference between baseline and 1 month after the third ranibizumab injection (0.07 ± 0.12) was not statistically significant (*p*=0.114, Wilcoxon signed-rank test) (Figure 2).

### 3.2. Secondary Efficacy Variables

**Figure 3:**
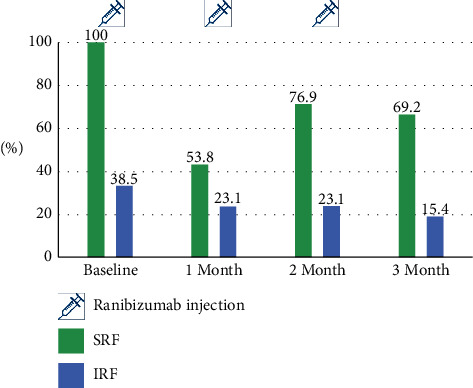
The proportion of eyes with subretinal fluid (SRF) and intraretinal fluid (IRF) after “switching.”

**Figure 4:**
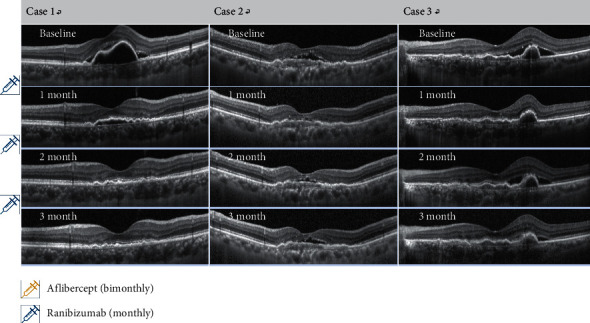
OCT findings of the typical cases showing a short-term anatomical good response after “switching” to monthly ranibizumab. One month after the first intravitreal ranibizumab injection, subretinal fluid (SRF) was dramatically decreased, and in 75%, it was completely absorbed. In 33.3%, no SRF recurred during 3 months after switching, as in case 1. Despite the consecutive monthly ranibizumab treatment, SRF recurrence was observed after the second intravitreal ranibizumab injection, as in cases 2 and 3. However, after 3 consecutive monthly ranibizumab injections, the height of the recurrent SRF was less than that of the baseline, and the final average CRT shows a statistically significant decrease compared to the baseline.

SRF was observed in the baseline OCT in all enrolled eyes. At “1 month” (1 month after the 1^st^ ranibizumab injection), the proportion of eyes with SRF decreased dramatically. In 53.8% of the enrolled eyes, SRF was completely absorbed, and 46.2% of the enrolled eyes showed residual SRF. However, despite continuing monthly ranibizumab injection, the tendency of SRF recurrence was observed at 1 month after second and third ranibizumab treatment. The height of the recurrent SRF was not higher than that of the baseline (Figures 3 and 4). Residual IRF was observed in 38.5% of the enrolled eye at baseline. The proportion of eyes with residual IRF gradually decreased to 15.4% at 1 month after the third ranibizumab injection.

### 3.3. Long-Term Maintenance of Treatment after Switching

**Figure 5:**
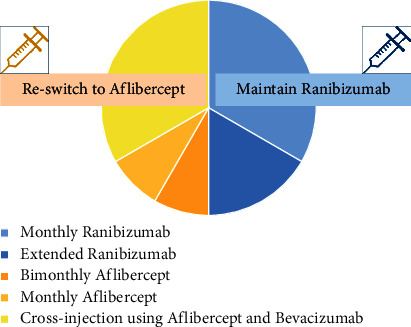
Treatment patterns 1 year after switching.

The long-term efficacy was assessed in 12 eyes that had been followed up for 1 year after switching. Among the 12 eyes, 6 eyes (50%) maintained ranibizumab treatment for 1 year, and the remaining 6 (50%) were reswitched to aflibercept-based treatment due to an insufficient response to monthly ranibizumab or patients' unwillingness to maintain monthly treatment. Of the 6 eyes treated with ranibizumab, 4 maintained monthly ranibizumab for 1 year and 2 extended the injection interval. The remaining six eyes were changed to an aflibercept-based regimen during the 1 year follow-up period. At the last visit, four of them were cross-injected with aflibercept and bevacizumab every month. One eye maintained bimonthly aflibercept, and the other maintained monthly aflibercept. The average number of monthly ranibizumab maintenance for 1 year after switching was 8.67 ± 2.90 (Figure 5).

## 4. Discussion

Switching to monthly ranibizumab with three consecutive injections in patients with nAMD showing an insufficient response to bimonthly aflibercept led to immediate anatomical improvement, especially in the first month after switching. Two months after switching, CRT and SRF tended to increase slightly again, indicating the switching effect's decay after repeated injections.

There have been several reports comparing the therapeutic effects of ranibizumab and aflibercept in treatment-naïve patients with nAMD. In these studies, equivalent therapeutic effects (in terms of functional and morphologic outcomes) and a similar injection burden were observed between ranibizumab and aflibercept [17–19]. It is also well known that monthly or bimonthly intravitreal aflibercept injections demonstrated similar therapeutic effects as monthly intravitreal ranibizumab injections [5]. In PCV, aflibercept treatment was more effective than ranibizumab for polyp regression [19].

**Figure 6:**
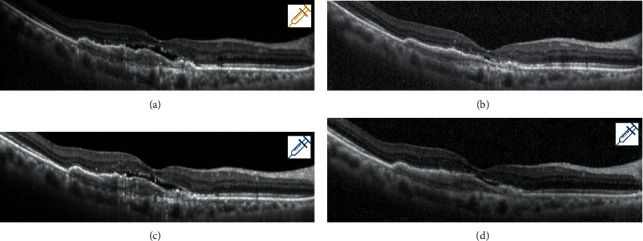
Treatment response 1 month after last aflibercept injection (1 month before “switching” to ranibizumab). (a) The OCT findings at the time of the last aflibercept injection. (b) The OCT findings 1 month after the last aflibercept injection. (c) The OCT findings at the time of the first ranibizumab injection after “switching.” (d) The OCT finding 1 month after the first ranibizumab injection. (b) shows a favorable anatomical outcome 1 month after the aflibercept injection, which means that not only the drug itself but also the injection interval affects the therapeutic effect of anti-VEGF agents in nAMD.

Apart from comparing anti-VEGF agents in patients with treatment-naïve nAMD, there have been studies related to the treatment response after anti-VEGF agent switching. A minority of patients with nAMD experience suboptimal treatment response to continued therapy with the same anti-VEGF agent (anti-VEGF resistance) [20–23]. Since aflibercept was introduced a few years later than ranibizumab and showed a higher affinity for VEGF, there have been many reports about the therapeutic effects of switching from ranibizumab to aflibercept [9–14]. Bakall et al. [10] and Grewal et al. [11] reported anatomical improvement after switching to aflibercept in patients with nAMD resistance to other anti-VEGF agents, bevacizumab and ranibizumab. However, potential factors explaining this favorable outcome after switching include tachyphylaxis, the natural course of the disease, and injection pattern, as well as the difference in the anti-VEGF agent itself. One of enrolled patients visited the clinic one month after the last aflibercept treatment and showed a favorable anatomical outcome 1 month before “switching,” which means that not only the drug itself but also the injection interval affects the therapeutic effect of anti-VEGF agents in nAMD (Figure 6).

Few studies have been conducted on the therapeutic effects of reverse switching from aflibercept to ranibizumab. The therapeutic effect of switching back to bevacizumab or ranibizumab for recurrent neovascular activity with aflibercept in nAMD was reported to be initially effective [15]. Recently, Gale et al. [8] suggested that patients with nAMD who have shown a suboptimal response to aflibercept may benefit from switching to ranibizumab. This study confirmed that the switching from aflibercept to ranibizumab led to a significant improvement in CRT, with approximately 60% experiencing stabilized or improved best-corrected visual acuity (BCVA), although this was counterbalanced by a large minority (38%) who lost ≥1 letter. The most remarkable improvement in clinical outcomes was observed during the initial monthly treatment period.

Our findings concur with these previous reports and suggest that the favorable treatment outcome of switching from bimonthly aflibercept to monthly ranibizumab might be related to anti-VEGF resistance. In addition, in our study, a group of patients had BCVA improved, and the change in average BCVA was not statistically significant but showed a tendency to improve after 3 monthly injections of ranibizumab compared to baseline. According to many previous studies about nAMD and visual outcomes, IRF had a much greater negative impact on the BCVA than SRF [24], while the presence of subfoveal SRF had little impact on mean visual acuity, and those with persistent SRF also seemed to maintain their vision in the long-term [25, 26]. Although the thickness of the retina gradually increased from 1 month after switching, it is possible that the final BCVA, 3 months after the switching point, improved due to the gradual absorption of IRF.

The results of “Long-term maintenance of treatment after switching” show that in 50%, the treatment results of ranibizumab were satisfactory, and the other 50% was reswitched due to the decay in the treatment response or the patient could not maintain the monthly injections. Therefore, although the short-term effect of switching to monthly ranibizumab showed a favorable response in the refractory cases, the efficacy tended to reduce gradually, and the long-term maintenance of monthly ranibizumab treatment might not be feasible in the real-world situation.

As many new anti-VEGF agents with longer half-lives are continuously being developed, it is important to elucidate the differences in therapeutic effects between different anti-VEGF agents. Because new anti-VEGF agents can be injected not only to treatment-naïve patients but also to patients treated with existing agents, studies about the clinical effectiveness of anti-VEGF resistance and the switching effect between various anti-VEGF agents should be conducted. Monthly aflibercept treatment and combination of aflibercept and ranibizumab or bevacizumab can be appropriate treatment options for some patients. In addition, a shift to new anti-VEGF agents, such as brolucizumab, which are expected to be more effective in nAMD refractory to conventional anti-VEGF agents, can be an alternative option [27].

The effectiveness of switching can be evaluated by CRT changes in the short-term and the reswitching rate in the long-term. In order to determine the characteristics of patients with poor treatment response or anti-VEGF resistance, we evaluated the age, subtype of nAMD, and the type or total number of anti-VEGF injections, especially previous ranibizumab injections. However, we observed no significant correlations. Furthermore, well-designed prospective or comparative studies are warranted to identify the long-term effect of switching, the duration of single anti-VEGF agent treatment before anti-VEGF resistance, and the selection of ideal anti-VEGF agents. In addition, a unified criterion of treatment failure for a specific anti-VEGF agent that can be used as the basis for regimen change should be established.

This study has several limitations. First, it is a retrospective, noncomparative case series that included a small number of cases. Moreover, there was no comparative data with monthly aflibercept treatment because of the Korean National Health Insurance System's restriction. Therefore, it is impossible to determine whether switching is due to anti-VEGF agents' change or injection schedules. We enrolled patients with nAMD refractory to current aflibercept treatment regardless of the treatment history (anti-VEGF agents, number of intravitreal anti-VEGF injections, PDT). Nevertheless, these patients can better represent patients with refractory nAMD in the real world. We closely followed the retinal morphology based on OCT at each visit. As a result, although the number of enrolled patients was not large and the treatment history was heterogeneous, the short-term treatment effect of switching to monthly ranibizumab was confirmed in enrolled patients.

## 5. Conclusion

In conclusion, monthly ranibizumab treatment might be an effective treatment option for patients with nAMD showing insufficient bimonthly aflibercept treatment. It should be more effective and useful in many countries, including Korea, where the national or private health insurance system limits the minimum interval of aflibercept injections as two months.

## Figures and Tables

**Table 1:** Clinical and demographic characteristics of included patients with neovascular age-related macular degeneration showing an insufficient response to bimonthly aflibercept.

Characteristic	Mean or number
Male-to-female ratio, no. (%)	11 : 2 (84.6% : 15.4%)
Mean age at baseline (y)	72.77 ± 8.27
Type of nAMD, typical nAMD : PCV (%)	7 : 6 (53.8% : 46.2%)
Average number of previous anti-VEGF injections	
Aflibercept	8.08 ± 4.97
Ranibizumab	2.00 ± 4.10
Bevacizumab	5.38 ± 11.1
Previous PDT (%)	2 (15.4%)
Baseline central retinal thickness, *μ*m	349.62 ± 223.51
Baseline best-corrected visual acuity, logMAR	0.50 ± 0.23

nAMD, neovascular age-related macular degeneration; PCV, polypoidal choroidal vasculopathy; R, right; L, left; A, aflibercept; B, bevacizumab; PDT, photodynamic therapy.

## Data Availability

The study contains the aggregated results extracted through encrypted data. It is difficult to disclose other details according to IRB regulations. If there is a critical issue, data are available through the reapproval process of the IRB and the corresponding author upon request.
